# Structural and Functional Brain Connectivity Changes Between People With Abdominal and Non-abdominal Obesity and Their Association With Behaviors of Eating Disorders

**DOI:** 10.3389/fnins.2018.00741

**Published:** 2018-10-11

**Authors:** Bo-yong Park, Mi Ji Lee, Mansu Kim, Se-Hong Kim, Hyunjin Park

**Affiliations:** ^1^Department of Electrical and Computer Engineering, Sungkyunkwan University, Suwon, South Korea; ^2^Center for Neuroscience Imaging Research, Institute for Basic Science, Suwon, South Korea; ^3^Departments of Neurology, Samsung Medical Center, Sungkyunkwan University School of Medicine, Seoul, South Korea; ^4^Department of Family Medicine, St. Vincent’s Hospital, Catholic University College of Medicine, Suwon, South Korea; ^5^School of Electronic and Electrical Engineering, Sungkyunkwan University, Suwon, South Korea

**Keywords:** abdominal obesity, multimodal imaging analysis, probabilistic fiber tractography, static and dynamic connectivity analysis, eating disorder behaviors

## Abstract

Abdominal obesity is important for understanding obesity, which is a worldwide medical problem. We explored structural and functional brain differences in people with abdominal and non-abdominal obesity by using multimodal neuroimaging and up-to-date analysis methods. A total of 274 overweight people, whose body mass index exceeded 25, were enrolled in this study. Participants were divided into abdominal and non-abdominal obesity groups using a waist–hip ratio threshold of 0.9 for males and 0.85 for females. Structural and functional brain differences were assessed with diffusion tensor imaging and resting-state functional magnetic resonance imaging. Centrality measures were computed from structural fiber tractography, and static and dynamic functional connectivity matrices. Significant inter-group differences in structural and functional connectivity were found using degree centrality (DC) values. The associations between the DC values of the identified regions/networks and behaviors of eating disorder scores were explored. The highest association was achieved by combining DC values of the cerebral peduncle, anterior corona radiata, posterior corona radiata (from structural connectivity), frontoparietal network (from static connectivity), and executive control network (from dynamic connectivity) compared to the use of structural or functional connectivity only. Our results demonstrated the effectiveness of multimodal imaging data and found brain regions or networks that may be responsible for behaviors of eating disorders in people with abdominal obesity.

## Introduction

Obesity is a worldwide medical problem that is responsible for inducing insulin resistance, type 2 diabetes, cardiovascular diseases, and some cancers (Raji et al., 2010; Malik et al., 2013; Val-Laillet et al., 2015). However, medical complications are not always manifested in people with obesity. A recent study showed that abdominal obesity was associated with increased risk for cardiovascular disease and mortality, while non-abdominal obesity had fewer adverse effects overall, and in some instances it even elicited protective effects (Zhang et al., 2008). In this context, the concept of metabolically healthy obesity has been extensively accepted (Stefan et al., 2013). In addition, previous studies suggested that abdominal obesity differs from non-abdominal obesity (Folsom et al., 1993, 2000; Bujalska et al., 1997; Després and Lemieux, 2006; Després et al., 2008). People with abdominal obesity have been shown to be at a higher risk for the metabolic syndrome, which is linked to diabetes and cardiovascular disease (Folsom et al., 2000; Després and Lemieux, 2006; Després et al., 2008). Folsom et al. (1993, 2000) found that abdominal obesity was a better biomarker for predicting mortality than non-abdominal obesity, and Bujalska et al. (1997) demonstrated that the risk of diabetes was better quantified with the waist–hip ratio (WHR), a measure of abdominal obesity, than with the body mass index (BMI), a measure of general obesity. These studies collectively provided a rationale for distinguishing abdominal obesity from non-abdominal obesity. Thus, identifying differences between abdominal and non-abdominal obesity may provide additional information for the better understanding of the diverse characteristics of obesity.

Obesity is a heterogeneous disease with a multifactorial etiology, including the eating behavior, and genetic and other environmental factors (Brownell and Wadden, 1991; McLaughlin, 2012). Binge eating behavior is believed to be genetically determined and is an important factor in obesity (Bulik et al., 2003; Carnell et al., 2011). However, only few prior studies have reported an association between dietary patterns and abdominal obesity (Azadbakht and Esmaillzadeh, 2011; Zhang et al., 2015). Correspondingly, the elucidation of the mechanisms based on which adverse eating behaviors may differentially affect the brain of the people with abdominal and non-abdominal obesity is largely unknown, and thus constitutes one of the aims of this study.

Recent studies reported that abdominal obesity is linked to altered reward and cognitive systems (Hollmann et al., 2012; Yates et al., 2012; Yau et al., 2012; Val-Laillet et al., 2015; Gaudio et al., 2017; Olivo et al., 2017), which regulate the appetite response (Hollmann et al., 2012; Val-Laillet et al., 2015). It has been shown that the altered reward and cognitive processes are highly associated with errant eating behaviors (Hollmann et al., 2012; Yates et al., 2012; Yau et al., 2012; Val-Laillet et al., 2015). In addition to brain function alterations, structural abnormalities were identified in people with abdominal obesity, such as altered fractional anisotropy and volume changes in white matter (Jagust et al., 2005; Gaudio et al., 2017; Olivo et al., 2017). These studies collectively suggest that abdominal obesity is possibly related to both brain structure and function. They also motivated us to consider multimodal neuroimaging to explore the differences between people with abdominal and non-abdominal obesity.

Many neuroimaging studies have used connectivity analysis to quantify brain structure and function (Bullmore and Sporns, 2009; Rubinov and Sporns, 2010). Connectivity analysis measures the connectedness among brain regions or networks. In this study, we performed connectivity analysis based on graph theory that requires graph nodes (i.e., brain regions or networks) and edges (i.e., neuronal fibers or correlation of time series data between nodes) based on many approaches (Bullmore and Sporns, 2009; Rubinov and Sporns, 2010). Connectivity analysis assesses the entire brain as a complex interconnected network, and many studies have successfully used connectivity analysis to explore Alzheimer’s disease, attention deficit/hyperactivity disorders, and schizophrenia (Seo et al., 2013; Damaraju et al., 2014; Park et al., 2016a).

Recently, neuroimaging methods, including magnetic resonance imaging (MRI) and positron emission tomography, have been used to non-invasively link obesity with brain structure and function (Tataranni et al., 1999; Jagust et al., 2005; Hollmann et al., 2012; Lips et al., 2014; Val-Laillet et al., 2015). However, almost all of the neuroimaging studies compared people with obesity to those with healthy weights (Jagust et al., 2005; Hollmann et al., 2012; Val-Laillet et al., 2015). Studies exploring the brain structure and function of people with abdominal and non-abdominal obesity are lacking, even though the two groups may have distinct brain structures and functions. To fill this gap, we aimed to compare structural and functional brain connectivity between abdominal and non-abdominal obesity groups by applying network analysis using diffusion tensor imaging (DTI) and resting-state functional MRI (rs-fMRI). Motivated by the recent findings of functional network changes and their associations with eating behaviors in people with obesity (Park et al., 2016b), we aimed to associate identified neuroimaging findings of abdominal obesity with eating behaviors.

## Materials and Methods

### Imaging Data and Participants

T1-weighted structural data, DTI, and rs-fMRI data were obtained from the openly accessible Nathan Kline Institute-Rockland Sample (NKI-RS) database (Nooner et al., 2012). All imaging data were scanned with a 3T Siemens Magnetom Trio Tim scanner. The scanning parameters for the T1-weighted structural data acquisitions were as follows: repetition time (TR) = 1,900 ms, echo time (TE) = 2.52 ms, flip angle = 9°, field-of-view (FOV) = 250 mm × 250 mm, 1 mm^3^ voxel resolution, and 176 slices. The DTI parameters were as follows: TR = 2,400 ms, TE = 85 ms, flip angle = 90°, FOV = 212 mm × 180 mm, 2 mm^3^ voxel resolution, 64 slices, b-value = 1,500 s/mm^2^, and gradient = 137. The rs-fMRI parameters were as follows: TR = 645 ms, TE = 30 ms, flip angle = 60°, FOV = 222 mm × 222 mm, 3 mm^3^ voxel resolution, 40 slices, and 900 volumes. Of the 650 total participants, participants with a BMI of 25 or greater and those with full demographic information and eating disorder examination questionnaire (EDE-Q) scores were considered in this study. Participants with severe head motion were excluded (see the Section “Preprocessing of Rs-fMRI Data”). The selected 274 participants were divided into abdominal and non-abdominal obesity groups based on WHR. Participants with a WHR larger than 0.9 for males and 0.85 for females were classified in the abdominal obesity group, and the remaining participants were classified in the non-abdominal obesity group (World Health Organization, 2008). Detailed demographics are reported in **Table 1**. The Institutional Review Board (IRB) of Sungkyunkwan University approved this retrospective study. Our study was performed in full accordance with local IRB guidelines and informed consent was obtained from all participants.

**Table 1:** Participant demographics.

Parameter		Abdominal obesity (*n* = 152)	Non-abdominal obesity (*n* = 122)	*P*-value
Age		54.94 (17.23)	40.84 (19.09)	<0.001
Sex (Male:Female)		69:83	47:75	0.2527^∗^
BMI		31.37 (5.01)	29.84 (4.40)	0.0086
WHR	Male	0.98 (0.06)	0.84 (0.04)	<0.001
	Female	0.91 (0.05)	0.79 (0.05)	<0.001
EDE-Q-R		1.44 (1.40)	1.67 (1.56)	0.2053
EDE-Q-E		0.35 (0.69)	0.46 (0.86)	0.2495
EDE-Q-S		1.85 (1.41)	1.87 (1.50)	0.8925
EDE-Q-W		1.54 (1.19)	1.58 (1.31)	0.7843

*^∗^Chi-square test. BMI, body mass index; WHR, waist–hip ratio; EDE-Q-R, eating disorder examination questionnaire restraint; EDE-Q-E, eating disorder examination questionnaire eating concern; EDE-Q-S, eating disorder examination questionnaire shape concern; EDE-Q-W, eating disorder examination questionnaire weight concern.*

### Tractography and Connectivity Analysis of DTI Data

**FIGURE 1:**
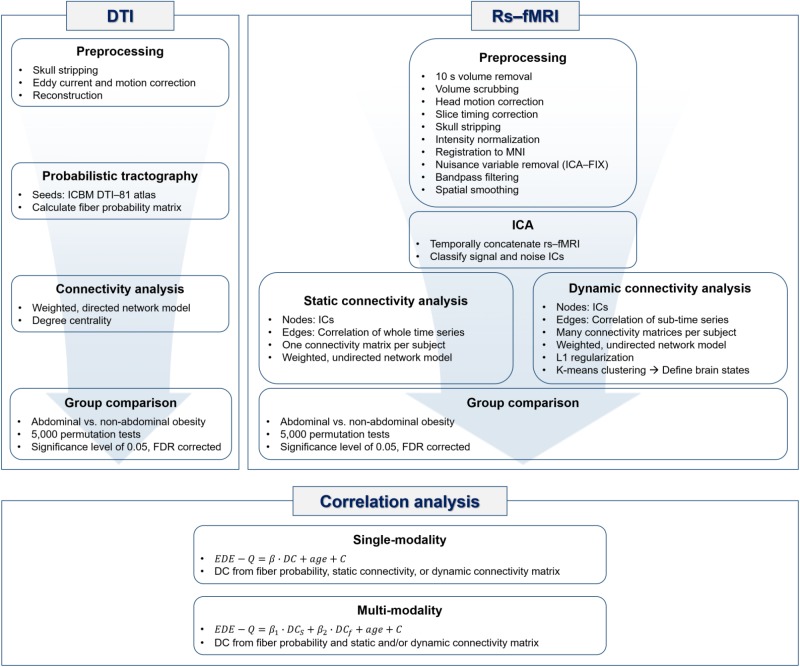
Flowchart of processing steps adopted in this analysis.

Probabilistic tractography of DTI data was performed using the FSL software (Jenkinson et al., 2012). The original DTI data were corrected for distortions due to eddy currents and head motion. DTI data were reconstructed based on the corresponding gradient table using the FDT toolbox. The diffusion parameters were computed with the Bedpostx toolbox, and probabilistic tractography was performed with the ProbtrackX toolbox in FSL (Jenkinson et al., 2012). Specifically, fibers were repeatedly sampled 5,000 times based on diffusion parameters that originated from the seed region and a probabilistic distribution of neuronal fibers was constructed. The probability of fibers connecting two brain regions was computed based on a probabilistic distribution of neuronal fibers, and the value was entered into a matrix called the fiber probability matrix. The nodes of the fiber probability matrix (i.e., brain regions) were defined by the ICBM DTI-81 atlas. A weighted and directed network model was applied to the fiber probability matrix and degree centrality (DC) values were computed. DC values were defined in a directed network model as the sum of in-degree and out-degree values that, respectively, included the column and row sums of the edge weights connected to the given node in the matrix (Rubinov and Sporns, 2010). DC values represent the importance of the node (Bullmore and Sporns, 2009; Rubinov and Sporns, 2010). DC values were used to identify brain regions that differed significantly between people with abdominal and non-abdominal obesity. The group differences in DC values between people with abdominal and non-abdominal obesity were assessed using permutation tests followed by false discovery rate (FDR) procedure to avoid multiple comparisons issues (Benjamini and Hochberg, 1995; Chen et al., 2013; Smith et al., 2013). We randomly assigned participants to either the abdominal or non-abdominal obesity group 5,000 times and created a null distribution of differences in DC values. If the differences in DC values of a region did not belong to the 95% of the null distribution, the region was considered significant. The *p*-values were further corrected using FDR (*p* < 0.05, corrected) (Benjamini and Hochberg, 1995). The overall processing flow of the DTI data is represented in the DTI part of **Figure 1**.

### Preprocessing of Rs-fMRI Data

Rs-fMRI data were preprocessed using the AFNI and FSL software (Cox, 1996; Jenkinson et al., 2012). The volumes of the first 10 s were discarded to adjust for the hemodynamic response delay. The frame-wise displacement (FD) between fMRI time series volumes was calculated and the volumes whose FDs exceeded 0.5 mm were eliminated (Power et al., 2012). We excluded participants who had more than 10% of the time series volumes removed. Volumes were adjusted for head motion and slice timing. The skull was removed, and the intensity was normalized with the mean value of 10,000 across 4D volumes. Rs-fMRI data were registered onto the T1-weighted structural data, followed by a subsequent registration onto the Montreal Neurological Institute (MNI) brain template. From the registered rs-fMRI data, nuisance variables of cerebrospinal fluid, white matter, head motion, cardiac, and large-vein-related artifacts were removed using the FMRIB’s ICA-based X-noiseifier (FIX) software (Salimi-Khorshidi et al., 2014). The first step of ICA-FIX performed a single independent component analysis (ICA) on an individual rs-fMRI data. From the decomposed independent components (ICs), a large number of temporal and spatial features were calculated. The calculated features were then input into a multilevel classifier, and signal and noise ICs were distinguished using the pretrained data by the Human Connectome Project (Salimi-Khorshidi et al., 2014). The noise ICs were regressed out from the rs-fMRI time series. A bandpass filter with a frequency between 0.009 and 0.08 and spatial smoothing with a full-width-at-half-maximum of 6 mm were applied.

### Group ICA

The rs-fMRI data from all participants were temporally concatenated, and group ICA was performed using the FSL MELODIC software (Beckmann et al., 2005; Jenkinson et al., 2012). The ICA produces spatial ICs by using probabilistic principal component analysis (Beckmann and Smith, 2004; Beckmann et al., 2005; Minka, 2000; Smith et al., 2012). The generated ICs were classified into signal or noise components according to two criteria. First, the ICs were compared with known resting state networks (RSNs) by using a cross-correlation, and those lower than 0.2 were considered as noise (Smith et al., 2009). Second, signal and noise components were classified by visual inspection considering the spatial and temporal characteristics (Kelly et al., 2010; Griffanti et al., 2017). Signal components showed a large spatial overlap with gray matter and a low overlap with white matter, cerebrospinal fluid, and blood vessels. The time series of the signal components was a regular wave without notable spikes, and had a power spectrum of at least one low-frequency peak between 0.01 and 0.1 Hz (Kelly et al., 2010; Griffanti et al., 2017).

### Static and Dynamic Connectivity Analyses of fMRI Data

The static and dynamic connectivity analyses with weighted and undirected network models were performed to quantify the functional characteristics of rs-fMRI. Graph nodes were defined as functionally interpretable ICs, and graph edges were defined as the Pearson correlation of time series between two nodes. For the static connectivity analysis, a connectivity matrix was constructed by computing the Pearson correlation of the entire time series between nodes, which yielded one connectivity matrix for each participant. A soft thresholding approach was adopted to avoid binarizing edge weights with the use of Equation 1.

(1) $$w_{\mathrm{ij}}={\left(\frac{r_{\mathrm{ij}}+1}{2}\right)}^{\beta }$$

where *r_ij_* is the correlation coefficient between nodes *i* and *j* (Mumford et al., 2010; Schwarz and McGonigle, 2011). β was set to six to conform to an unsigned network (Mumford et al., 2010). The elements of the soft thresholded matrix were converted to *z*-values using the Fisher’s *r*-to-*z* transformation. DC values were extracted from the *z*-transformed matrix by summing the edge weights connected to the given node in the column direction of the matrix and were then used to compare groups. The differences in DC values between people with abdominal and non-abdominal obesity were assessed based on 5,000 permutations, and the *p*-values were corrected using FDR (*p* < 0.05, corrected).

The dynamic connectivity analysis used a sliding window technique (Allen et al., 2012; Damaraju et al., 2014). A window size of 172 TRs (111 s) and a stride of 1 TR (0.645 s) were used to capture the lowest frequency (0.009 Hz) of the data (Hutchison et al., 2013; Damaraju et al., 2014). A rectangular window was convolved with a Gaussian kernel of size three. Finally, many dynamic connectivity matrices (mean of 770.45 and a standard deviation [SD] of 26.14) were constructed. L1 regularization was applied to the matrices to avoid ill-posed problems due to the limited information on the short time series segments. The regularization parameter λ was optimized with a cross-validation framework by maximizing the following log-likelihood function (Equation 2).

(2) $$\log\det\theta -\mathrm{tr}(S\theta )-\lambda ||\theta |{|}_{1}$$

where θ is the precision matrix, *S* is the empirical covariance matrix, *tr* is the trace, and || .*||*_1_ is the L1 norm (Friedman et al., 2008; Allen et al., 2012; Damaraju et al., 2014). Regularized dynamic connectivity matrices were concatenated across participants and grouped into several clusters by a K-means clustering algorithm to define brain states (Allen et al., 2012; Damaraju et al., 2014). The number of clusters was determined using the silhouette coefficient and elbow method (Allen et al., 2012; Kodinariya and Makwana, 2013; Damaraju et al., 2014). The most common number of clusters was considered as the optimal number and was used for group-level clustering of the concatenated dynamic connectivity matrices. Using the determined number of clusters, participant-level states were defined by applying the K-means clustering algorithm for each participant. We started with the participant-level cluster that explained the largest variance, and we matched it with the group-level state that yielded the maximum Pearson’s correlation (Park et al., 2018). We repeated the process for the subsequent participant-level cluster with the largest variance explained excluding the clusters that had already been processed. This allowed us to map each participant-level state with a corresponding group-level state. The dynamic connectivity matrices of each state were averaged, and DC values were extracted from the mean state matrices for each participant. The brain networks with significant differences in DC values between people with abdominal and non-abdominal obesity were assessed based on the 5,000 permutations followed by FDR at a significance level of 0.05. The overall rs-fMRI processing steps are summarized in the rs-fMRI part of **Figure 1**.

### Representative Brain States

Group-level states were defined from the dynamic connectivity analysis, and each state was associated with brain networks by computing the hubness of nodes for each state. For each group-level state, the betweenness centrality (BC) values of all nodes were calculated and normalized by dividing them with the mean value. Nodes with normalized BC values higher than 1.5 were defined as hub nodes, and were considered as a representative network for the state (Seo et al., 2013).

### Correlation Between DC Values and Behaviors of Eating Disorder Scores

DC values of the brain regions or networks that yielded significant group-wise differences in structural or functional connectivity between abdominal and non-abdominal obesity were correlated with behaviors of eating disorders assessed with the EDE-Q (Fairburn and Beglin, 1994; Mond et al., 2004). The EDE-Q assessment contained four subscales of restraint (EDE-Q-R), eating concern (EDE-Q-E), shape concern (EDE-Q-S), and weight concern (EDE-Q-W), which were based on the self-reported questionnaire (Fairburn and Beglin, 1994; Mond et al., 2004). A multiple linear regression model was constructed in accordance to Equation 3.

(3) EDE−Q=β⋅DC+age+C

where *DC* is the DC value of the brain region or network identified in the structural or functional connectivity analysis, β is the regression coefficient, and *C* is a constant. Age was added as a covariate to adjust for the difference between people with abdominal and non-abdominal obesity groups. A regression model was also constructed with DC values of brain regions and networks identified in both the structural and functional connectivity analyses in accordance to Equation 4.

(4) $$\mathrm{EDE}-Q=\beta_{1}\cdot {\mathrm{DC}}_{s}+\beta_{2}\cdot {\mathrm{DC}}_{f}+\mathrm{age}+C$$

where *DC_s_* denotes the DC values of the brain regions identified in the structural connectivity analysis, and *DC_f_* indicates the DC values of the brain networks identified in the functional connectivity analysis. The correlation analysis is summarized in the correlation analysis part of **Figure 1**.

### Statistics

The structural and functional group differences in DC values between people with abdominal and non-abdominal obesity were assessed using permutation tests followed by FDR. Participants were randomly assigned to the abdominal and non-abdominal obesity groups 5,000 times, and a null distribution was created. A brain region or network with DC values outside the 95% of the null distribution was considered to be associated with significant differences between the abdominal and non-abdominal obesity groups. The *p*-values were further corrected using the FDR approach suggested by Benjamini and Hochberg (*p* < 0.05, corrected) (Benjamini and Hochberg, 1995). The representative networks of group-level states in the dynamic connectivity analysis were defined using normalized BC values that were higher than 1.5. Correlation between DC values and EDE-Q scores were computed using a multiple linear regression model. The quality of the correlation was quantified using *R^2^* and *p*-values. The DC values of the identified brain regions or networks were correlated with four EDE-Q scores. The correlation results were corrected for the identified brain regions/networks, and for the four EDE-Q scores with FDR (Benjamini and Hochberg, 1995). All statistical analyses were performed in MATLAB (Mathworks Inc., Natick, MA, United States).

## Results

### Nodes for Connectivity Analysis

**FIGURE 2:**
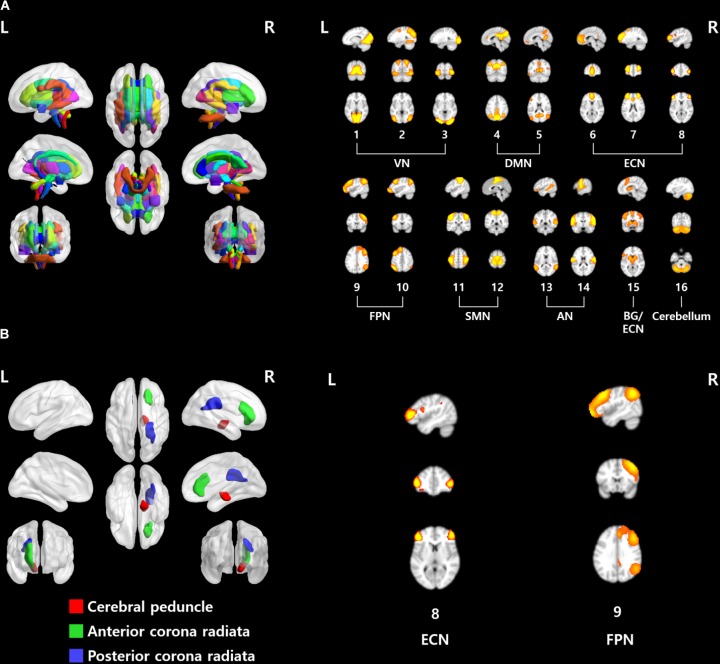
**(A)** ROIs used in this study. (Left) ICBM DTI-81 atlas and (right) 16 functionally interpretable ICs. **(B)** Brain regions and networks that showed significant associations with EDE-Q scores. VN, visual network; DMN, default mode network; ECN, executive control network; FPN, frontoparietal network; SMN, sensorimotor network; AN, auditory network; BG, basal ganglia.

The structural characteristics of the brain were quantified with a fiber probability matrix. The nodes of the fiber probability matrix were defined by the ICBM DTI-81 atlas (**Figure 2A**). Functional characteristics of the brain were quantified with rs-fMRI. Group ICA was performed to define brain networks. Twenty spatial ICs that explained 94.1% of the variance were automatically generated and four ICs were eliminated as noise components. The 16 functionally interpretable ICs (mean correlation with RSNs of *r* = 0.48, with an SD of 0.14) were considered to be graph nodes (**Figure 2A**). ICs 1–3 constitute the visual network (VN), ICs 4 and 5 constitute the default mode network (DMN), ICs 6–8 constitute the executive control network (ECN), ICs 9 and 10 constitute the frontoparietal network (FPN), ICs 11 and 12 constitute the sensorimotor network (SMN), ICs 13 and 14 constitute the auditory network (AN), IC 15 denotes the basal ganglia (BG) with part of the ECN, and IC 16 is the cerebellum.

### Differences in Structural and Functional Connectivity

We compared the structural brain differences between people with abdominal and non-abdominal obesity using the probabilistic fiber tractography approach derived from DTI. DC values, which represent the importance of a given node, were calculated from the fiber probability matrix. DC values were used to identify significant differences between groups. The pontine crossing tract, fornix, corticospinal tract, medial lemniscus, inferior and superior cerebellar peduncles, cerebral peduncle, internal capsule, anterior, superior, and posterior corona radiata, thalamic radiation, sagittal stratum, superior longitudinal fasciculus, and superior fronto-occipital fasciculus, showed significant between group differences (*p* < 0.05, permutation followed by FDR correction) (**Supplementary Table S1**).

**FIGURE 3:**
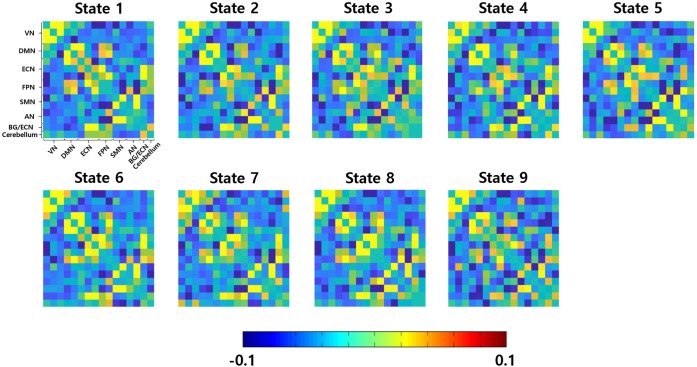
Nine group-level brain states. VN, visual network; DMN, default mode network; ECN, executive control network; FPN, frontoparietal network; SMN, sensorimotor network; AN, auditory network; BG, basal ganglia.

**FIGURE 4:**
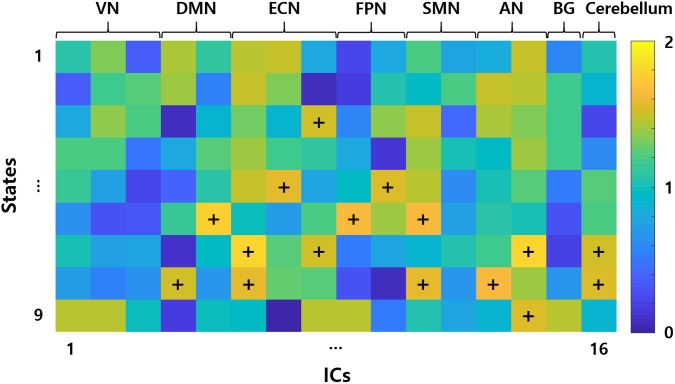
Representative networks of each group-level states. Elements of the matrix represent the hubness of BC. Representative networks are marked with the symbols “+.” VN, visual network; DMN, default mode network; ECN, executive control network; FPN, frontoparietal network; SMN, sensorimotor network; AN, auditory network; BG, basal ganglia; ICs, independent components.

The inter-group differences in the static and dynamic connectivity analyses were performed by using the 16 ICs as the graph nodes. One connectivity matrix was constructed for each participant for the static connectivity analysis. DC values were computed from the connectivity matrix and VN (ICs #1–3), DMN (IC #5), ECN (IC #6), FPN (ICs #9 and 10), and SMN (ICs #11 and 12), yielded significant differences in DC values between people with abdominal and non-abdominal obesity groups (**Supplementary Table S2**, *p* < 0.05, permutation followed by FDR correction). For the dynamic connectivity analysis, many connectivity matrices (mean of 770.45 and SD of 26.14) were constructed, and the matrices were grouped into nine clusters (i.e., brain states) using a K-means clustering algorithm (**Figure 3**). For each group-level state, the hubness of any node was computed, and if the given node satisfied the hub node criterion, it was considered as a representative network for the state (Seo et al., 2013). The ECN (IC #8, normalized BC 1.51) in state 3, ECN (IC #7, normalized BC 1.57), and FPN (IC #10, normalized BC 1.56) in state 5, DMN (IC #5, normalized BC 1.77), FPN (IC #9, normalized BC 1.63), and SMN (IC #11, normalized BC 1.63) in state 6, two ECNs (ICs #6 and 8, normalized BCs 1.81 and 1.52, respectively), AN (IC #14, normalized BC 1.79), and cerebellum (IC #16, normalized BC 1.51) in state 7, DMN (IC #4, normalized BC 1.52), ECN (IC #6, normalized BC 1.57), SMN (IC #11, normalized BC 1.57), AN (IC #13, normalized BC 1.63), and cerebellum (IC #16, normalized BC 1.55) in state 8, and AN (IC #14, normalized BC 1.54) in state 9, were identified as the representative networks (**Figure 4**). The connectivity matrices were clustered and averaged to yield a mean state matrix for each brain state. DC values were extracted from the mean state matrices, and the groups were then compared. VN (IC #2), DMN (IC #5), ECN (IC #6), FPN (ICs #9 and 10), and SMN (ICs #11 and 12), showed significant (*p* < 0.05, permutation followed by FDR correction) inter-group differences in DC values, and the results for each state were reported in the **Supplementary Table S3**.

### Correlation Between DC Values and Behaviors of Eating Disorder Scores

We calculated the correlation between the DC values of the identified brain regions from structural connectivity analysis or brain networks from the functional connectivity analysis and EDE-Q scores. The correlation between neuroimaging findings and the behaviors of eating disorders was explored as an eating disorder that was reported to be associated with abdominal obesity (Dallman et al., 2003; Gluck et al., 2004; Daubenmier et al., 2011; Succurro et al., 2015).

When only the estimated DC values from the structural connectivity analysis were used, the right cerebral peduncle, the right anterior, and posterior corona radiata were significantly correlated with EDE-Q scores (**Figure 2B** and **Table 2**, *p* < 0.05, FDR corrected). In the case of the static connectivity analysis, FPN (IC #9) yielded significant correlations with EDE-Q-S and W scores (**Figure 2B** and **Table 2**, *p* < 0.05, FDR corrected). In the case of the dynamic connectivity analysis, no networks showed significant correlation with EDE-Q scores at the significance level of 0.05. When we relaxed the significance level to 0.1, ECN (IC #8) in state 5 was correlated with the EDE-Q-S score (**Figure 2B** and **Table 2**, *p* < 0.1, FDR corrected).

**Table 2:** Brain regions and networks that yielded significant correlations between the DC values of the identified regions/networks and the EDE-Q scores.

Information	Region/Network	EDE-Q	*R^2^*	*P*-value
DTI	Right cerebral peduncle	E	0.0377	0.0109
		S	0.0324	0.0153
		W	0.0530	0.0025
	Right anterior corona radiata	R	0.0323	0.0468
	Right posterior corona radiata	R	0.0288	0.0407
		S	0.0283	0.0407
fMRI: Static	FPN (IC #9)	S	0.0270	0.0491
		W	0.0363	0.0268
fMRI: Dynamic	ECN (IC #8) in state 5	S	0.0285	0.0794

*The significance of the dynamic connectivity analysis was 0.1. P-values were corrected using FDR. DC, degree centrality; EDE-Q-R, eating disorder examination questionnaire restraint; EDE-Q-E, eating disorder examination questionnaire eating concern; EDE-Q-S, eating disorder examination questionnaire shape concern; EDE-Q-W, eating disorder examination questionnaire weight concern; FPN, frontoparietal network; ECN, executive control network.*

We performed further correlation analysis that combined the DC values identified from the single-modal results. We considered various combinations of the following DC values: the right cerebral peduncle and right anterior and posterior corona radiata from structural connectivity analysis, FPN (IC #9) from the static functional connectivity analysis, and the ECN (IC #8) in state 5 from the dynamic functional connectivity analysis (**Table 3**). Our results showed that EDE-Q scores elicited the highest correlations with the DC values that combined the identified brain regions and networks from the structural connectivity, static functional connectivity, and dynamic functional connectivity analyses, followed by the structural connectivity and dynamic functional connectivity analyses, the structural connectivity and static functional connectivity analyses, structural connectivity analysis only, static and dynamic functional connectivity analyses, static functional connectivity analysis only, and the dynamic functional connectivity analysis only.

**Table 3:** Correlation analysis between DC values of both the brain regions and networks that showed a good correlation in the first step and EDE-Q scores.

Information	EDE-Q							
	R		E		S		W	
	*R^2^*	*P*-value	*R^2^*	*P*-value	*R^2^*	*P*-value	*R^2^*	*P*-value
Only DTI	***0.0453***	***0.0139***	***0.0530***	***0.0054***	***0.0602***	***0.0022***	***0.0765***	***<0.001***
Only fMRI: Static	0.0130	0.1691	0.0113	0.2132	***0.0270***	***0.0245***	***0.0363***	***0.0067***
Only fMRI: Dynamic	0.0072	0.3777	0.0118	0.2005	***0.0285***	***0.0198***	0.0174	0.0921
fMRI: Static and dynamic	0.0183	0.1725	0.0195	0.1490	***0.0566***	***0.0013***	***0.0589***	***<0.001***
DTI and fMRI: Static	***0.0516***	***0.0139***	***0.0567***	***0.0077***	***0.0759***	***<0.001***	***0.1105***	***<0.001***
DTI and fMRI: Dynamic	***0.0497***	***0.0174***	***0.0609***	***0.0046***	***0.0868***	***<0.001***	***0.0953***	***<0.001***
DTI and fMRI: Static and dynamic	***0.0576***	***0.0140***	***0.0662***	***0.0053***	***0.1087***	***<0.001***	***0.1294***	***<0.001***

*P-values were corrected using FDR. Significant results (p < 0.05) are reported in bold italics. DC, degree centrality; EDE-Q-R, eating disorder examination questionnaire restraint; EDE-Q-E, eating disorder examination questionnaire eating concern; EDE-Q-S, eating disorder examination questionnaire shape concern; EDE-Q-W, eating disorder examination questionnaire weight concern.*

## Discussion

We explored the differences in structural and functional connectivity in brain regions and networks related to the behaviors of eating disorders between people with abdominal and non-abdominal obesity with the use of DTI and rs-fMRI. Probabilistic fiber tractography and static and dynamic connectivity analyses were used to quantify the characteristics of brain regions and networks. The DC values of several brain regions and networks showed significant inter-group differences. We further explored the relationship between structural and functional connectivity and key behaviors of eating disorders, and significant correlations were found. Our results indicated that altered brain structure and function in people with abdominal and non-abdominal obesity were associated with eating disorders behaviors.

We found that the functional connectivity in FPN and ECN showed significant inter-group differences between people with abdominal and non-abdominal obesity. Furthermore, significant associations with key behaviors of eating disorders were observed. The FPN and ECN mainly contain the dorsolateral prefrontal cortex which controls cognitive functions, such as planning, working memory, and inhibition (Le et al., 2007; Stice et al., 2008; Davids et al., 2010). Some considered obesity as a type of psychological disease related to deranged eating behaviors, which results from a dysfunctional fronto-striatal circuitry (Volkow et al., 2009). Previous studies observed that the impaired inhibitory control in people with obesity suggesting the lateral prefrontal cortex is an important region of the fronto-striatal circuit that controls the regulation of eating behaviors (DelParigi et al., 2004; Wang et al., 2004; Le et al., 2006). One study showed that altered activity in the dorsolateral prefrontal cortex broke the balance between the reward and cognitive systems and led to errant eating behaviors (Val-Laillet et al., 2015). In addition, previous studies found an imbalance in the prefrontal and limbic brain circuits that support aspects of cognition- and reward-related eating behaviors (Carnell et al., 2011; Brooks et al., 2013; Vainik et al., 2013). In summary, it is possible that lack of regulatory influences from the dorsolateral prefrontal cortex in people with obesity might cause a psychological dependence on food and overeating (Wang et al., 2004).

One possible interpretation of our findings on differences between people with abdominal and non-abdominal obesity is that insulin levels may influence brain function during rest in brain networks that control reward and food regulation. Abdominal obesity is known to be strongly related to insulin resistance, and it has been recognized as a key determinant of the metabolic syndrome (Carr et al., 2004; Després and Lemieux, 2006). A previous study reported that individuals who were resistant to insulin showed an increase of functional connectivity in the reward network, but a reduction in cognitive control networks (Kullmann et al., 2012). In addition, altered brain activity in the frontoparietal executive system was found in metabolic syndrome patients (Hoth et al., 2011). Interestingly, a study that utilized intranasal insulin administration reported altered brain activity in cognitive brain regions and altered functional connectivity between the hippocampal region and DMN (Kullmann et al., 2017). The change of functional connectivity in the hippocampal region was significantly correlated with visceral adipose tissue and the change in subjective feeling of hunger after intranasal insulin administration (Kullmann et al., 2017). These studies collectively suggest that abdominal obesity may be indirectly related to altered function in cognitive related brain areas.

In addition to functional connectivity differences, the inter-group structural connectivity differences were observed in many brain regions (**Supplementary Table S1**). Among them, the anterior and posterior corona radiata and the cerebral peduncle yielded significant associations with behaviors of eating disorders. The corona radiata is part of the limbic-thalamo-cortical circuitry that is critical for reward and cognitive processes (Catani et al., 2002; Kalivas and Volkow, 2005; Olivo et al., 2017). The corona radiata is a key region that projects thalamic information onto the prefrontal cortex, and the alteration in this region is shown to be associated with cognitive dysfunction and central taste disorders (Shott et al., 2015; Olivo et al., 2017). Previous studies found that fractional anisotropy in corona radiata showed abnormalities in people with anorexia nervosa (Gaudio et al., 2017), and it was significantly related to the behaviors of eating disorders (Olivo et al., 2017). They also reported that altered white matter integrity in corona radiata was associated with dysfunctions in reward and cognitive related processes, which led to eating disorders (Olivo et al., 2017). Our results and these studies were consistent in that altered structural connectivity in corona radiata was related to the behaviors of eating disorders. In addition to the corona radiata, we found significant inter-group structural connectivity differences in the cerebral peduncle and associations with the behaviors of eating disorders. The cerebral peduncle is included in the corticospinal tracts that contain large fiber tracts (Ramnani, 2006; Koyama et al., 2013). The fiber tracts of the cerebral peduncle primarily originate from the prefrontal cortex in the human (Ramnani, 2006). The prefrontal cortex controls the process of reward, inhibitory control, and executive decision making (Holland and Gallagher, 2004; Kringelbach and Rolls, 2004; Petrovich et al., 2007). It is an important region that modulates an individual’s eating behavior (van Vugt, 2009). Previous studies observed altered white matter integrity of the cerebral peduncle, including the cortico-spinal and cortico-bulbar tracts in people with obesity (Civardi et al., 2004; Karlsson et al., 2013; Ryan and Walther, 2014; van Bloemendaal et al., 2016; Papageorgiou et al., 2017). Taken together, the structural connectivity in the cerebral peduncle, which is strongly connected to the prefrontal cortex, may be indirectly related to eating behaviors in people with obesity. However, the cerebral peduncle contains fiber tracts that originate from the motor, temporal, and parietal cortices, as well as the prefrontal cortex (Ramnani, 2006). Thus, additional experiments are required to elucidate the relationship between structural connectivity in the cerebral peduncle and eating behaviors in more detail.

In the correlation analyses conducted herein, we found that the DC values computed from multimodality imaging data (i.e., both DTI and rs-fMRI) explained the behaviors of eating disorders with higher *R*^2^ values compared to those from single-modality imaging data (i.e., DTI or rs-fMRI alone) (**Table 3**). This result may be attributed to the heterogeneity of obesity associated with eating behaviors, genetic factors, or insulin resistance (Brownell and Wadden, 1991; McLaughlin, 2012). Using only structural or functional characteristics of the brain may not provide sufficient information to quantify the diverse aspects of obesity. The results indicate that multimodality imaging data provides complementary information to understand the links between the brain and behaviors of eating disorders. In this study, both static and dynamic functional connectivity analyses were considered, and the dynamic connectivity results correlated better with behaviors of eating disorders. Unlike the static connectivity analysis, many connectivity matrices that reflect the temporal dynamics of the brain states were constructed in the dynamic connectivity analysis. The additional information may better link changes in the brain structure and function to behaviors associated with eating disorders. Our study suggested that using the structural and functional information and using dynamic, rather than static, connectivity analysis could best explain the elicited behaviors of eating disorders.

Our study has a few limitations. Although we associated brain states derived from dynamic connectivity analysis with representative brain networks, we could not match the brain states with specific cognitive conditions owing to validation difficulties. In future studies, we will collect various clinical scores to correlate cognitive conditions with brain states. In this study, we used WHR instead of other direct measures of abdominal obesity, such as visceral fat from abdominal MRI and body fat measures from bioelectrical impedance analyses. The NKI-RS database did not provide such measures, and we were thus limited to the use of WHR.

In this study, we found significant brain structural and functional differences between people with abdominal and non-abdominal obesity and strong associations between the connectivity values and EDE-Q scores in the cerebral peduncle, anterior and posterior corona radiata, ECN, and FPN. When both the structural and dynamic functional connectivity were used, the relationships between brain connectivity and the behaviors of the eating disorders were strengthened, thus indicating that multimodal imaging data is more effective than single-modal imaging data. Our reported results are expected to provide more evidence on the mechanisms associated with abdominal obesity and behaviors of eating disorders.

## Data Availability Statement

The imaging and phenotypic dataset analyzed for this study can be found in the enhanced NKI-RS database (http://fcon_1000.projects.nitrc.org/indi/enhanced/). Interested researchers can contact the administrator of this database to request access to the data.

## Author Contributions

BP and HP wrote the manuscript. MK aided the experiments. ML and SK aided the clinical interpretation. HP is the guarantor of this work, and as such, had full access to all the data in the study and takes responsibility for the integrity of the data and the accuracy of the data analyses.

## Conflict of Interest Statement

The authors declare that the research was conducted in the absence of any commercial or financial relationships that could be construed as a potential conflict of interest.
