# Supplementary material for: Structural and Functional Brain Connectivity Changes Between People With Abdominal and Non-abdominal Obesity and Their Association With Behaviors of Eating Disorders
Source: Front Neurosci. 2018 Oct 11;12:741. doi: 10.3389/fnins.2018.00741 (PMC6193119; doi:10.3389/fnins.2018.00741)
Supplement: Supplementary file 1 [file Table_1.docx]

Supplementary Material

Structural and functional brain connectivity changes between people with abdominal and non-abdominal obesity and their association with behaviors of eating disorders

Bo-yong Park,^1,2^ Mi Ji Lee,^3^ Mansu Kim,^1,2^ Se-Hong Kim^4^, and Hyunjin Park^2,5*^

*** Correspondence:** Hyunjin Park: hyunjinp@skku.edu

# Supplementary Tables

**Supplementary Table S1.** Brain regions that showed significant inter-group differences in DC values calculated from the fiber probability matrix between people with abdominal and non-abdominal obesity. *P*-values were corrected using permutation followed by FDR. Regions that showed significant (*p* < 0.05) associations with EDE-Q scores are reported in bold italics.

| Region | *p*-value, corrected |
| --- | --- |
| Pontine crossing tract | 0.0117 |
| Fornix (column and body) | 0.0400 |
| Corticospinal tract right | 0.0365 |
| Corticospinal tract left | 0.0223 |
| Medial lemniscus left | 0.0325 |
| Inferior cerebellar peduncle right | 0.0117 |
| Inferior cerebellar peduncle left | 0.0150 |
| Superior cerebellar peduncle left | 0.0325 |
| ***Cerebral peduncle right*** | ***0.0400*** |
| Posterior limb of internal capsule right | 0.0353 |
| Retrolenticular part of internal capsule right | 0.0467 |
| ***Anterior corona radiata right*** | ***0.0117*** |
| Anterior corona radiata left | 0.0117 |
| Superior corona radiata right | 0.0117 |
| ***Posterior corona radiata right*** | ***0.0358*** |
| Posterior corona radiata left | 0.0117 |
| Posterior thalamic radiation right | 0.0150 |
| Posterior thalamic radiation left | 0.0150 |
| Sagittal stratum right | 0.0200 |
| Sagittal stratum left | 0.0200 |
| Fornix (cres) / Stria terminalis right | 0.0358 |
| Superior longitudinal fasciculus right | 0.0325 |
| Superior fronto-occipital fasciculus right | 0.0150 |
| Superior fronto-occipital fasciculus left | 0.0400 |

DC, degree centrality; EDE–Q, eating disorder examination questionnaire

**Supplementary Table S2.** Brain networks that showed significant inter-group differences in DC values calculated from the static connectivity matrix between people with abdominal and non-abdominal obesity. *P*-values were corrected using permutation followed by FDR. Networks that showed significant (*p* < 0.05) associations with EDE-Q scores are reported in bold italics.

| Network | *p*-value, corrected |
| --- | --- |
| VN (IC #1) | 0.0261 |
| VN (IC #2) | 0.0261 |
| VN (IC #3) | 0.0080 |
| DMN (IC #5) | 0.0462 |
| ECN (IC #6) | 0.0261 |
| ***FPN (IC #9)*** | ***0.0261*** |
| FPN (IC #10) | 0.0080 |
| SMN (IC #11) | 0.0171 |
| SMN (IC #12) | 0.0352 |

DC, degree centrality; EDE–Q, eating disorder examination questionnaire; VN, visual network; DMN, default mode network; ECN, executive control network; FPN, frontoparietal network; SMN, sensorimotor network.

**Supplementary Table S3.** Brain networks that showed significant inter-group differences in DC values calculated from the dynamic connectivity matrix between people with abdominal and non-abdominal obesity. *P*-values were corrected using permutation followed by FDR. Networks that showed good (*p* < 0.1) associations with EDE-Q scores are reported in bold italics.

| Network | | *p*-value, corrected |
| --- | --- | --- |
| State 1 | DMN (IC #5) | 0.0288 |
|  | SMN (IC #11) | 0.0496 |
| State 2 | ECN (IC #6) | 0.0247 |
|  | FPN (IC #9) | < 0.001 |
| State 3 | ECN (IC #6) | 0.0354 |
| State 4 | FPN (IC #9) | 0.0288 |
| State 5 | ***ECN (IC #8)*** | ***0.0354*** |
|  | FPN (IC #9) | 0.0247 |
|  | FPN (IC #10) | 0.0247 |
|  | SMN (IC #12) | 0.0378 |
| State 6 | VN (IC #2) | 0.0378 |
|  | ECN (IC #6) | 0.0247 |
| State 7 | ECN (IC #6) | 0.0288 |
|  | FPN (IC #10) | 0.0288 |
| State 8 | FPN (IC #10) | 0.0378 |
|  | SMN (IC #12) | 0.0247 |
| State 9 | ECN (IC #6) | 0.0247 |
|  | SMN (IC #12) | 0.0440 |

DC, degree centrality; EDE–Q, eating disorder examination questionnaire; VN, visual network; DMN, default mode network; ECN, executive control network; FPN, frontoparietal network; SMN, sensorimotor network.
